# Genetic and Targeted eQTL Mapping Reveals Strong Candidate Genes Modulating the Stress Response During Chicken Domestication

**DOI:** 10.1534/g3.116.037721

**Published:** 2016-12-10

**Authors:** Amir Fallahsharoudi, Neil de Kock, Martin Johnsson, Lejla Bektic, S. J. Kumari A. Ubhayasekera, Jonas Bergquist, Dominic Wright, Per Jensen

**Affiliations:** * AVIAN Behavioural Genomics and Physiology Group, Department of Physics, Chemistry and Biology, Linköping University, 58183, Sweden; † Department of Chemistry, BMC, Analytical Chemistry and Neurochemistry, University of Uppsala, 75124, Sweden

**Keywords:** animal domestication, quantitative trait genes, corticosterone, aldosterone

## Abstract

The stress response has been largely modified in all domesticated animals, offering a strong tool for genetic mapping. In chickens, ancestral Red Junglefowl react stronger both in terms of physiology and behavior to a brief restraint stress than domesticated White Leghorn, demonstrating modified functions of the hypothalamic–pituitary–adrenal (HPA) axis. We mapped quantitative trait loci (QTL) underlying variations in stress-induced hormone levels using 232 birds from the 12th generation of an advanced intercross between White Leghorn and Red Junglefowl, genotyped for 739 genetic markers. Plasma levels of corticosterone, dehydroepiandrosterone (DHEA), and pregnenolone (PREG) were measured using LC-MS/MS in all genotyped birds. Transcription levels of the candidate genes were measured in the adrenal glands or hypothalamus of 88 out of the 232 birds used for hormone assessment. Genes were targeted for expression analysis when they were located in a hormone QTL region and were differentially expressed in the pure breed birds. One genome-wide significant QTL on chromosome 5 and two suggestive QTL together explained 20% of the variance in corticosterone response. Two significant QTL for aldosterone on chromosome 2 and 5 (explaining 19% of the variance), and one QTL for DHEA on chromosome 4 (explaining 5% of the variance), were detected. Orthologous DNA regions to the significant corticosterone QTL have been previously associated with the physiological stress response in other species but, to our knowledge, the underlying gene(s) have not been identified. *SERPINA10* had an expression QTL (eQTL) colocalized with the corticosterone QTL on chromosome 5 and *PDE1C* had an eQTL colocalized with the aldosterone QTL on chromosome 2. Furthermore, in both cases, the expression levels of the genes were correlated with the plasma levels of the hormones. Hence, both these genes are strong putative candidates for the domestication-induced modifications of the stress response in chickens. Improved understanding of the genes associated with HPA-axis reactivity can provide insights into the pathways and mechanisms causing stress-related pathologies.

Stress affects the welfare of billions of animals in food production worldwide. It may cause behavioral disorders and increase the risk of disease (Lupien *et al.* 2009), both of which have direct consequences for humans. For example, disease control in farm animals is an important contributor to the dramatic increase in antibiotic resistance, which in turn is one of the major threats to human health (Rostagno 2009). Hence, understanding the biological foundation of the stress response is of central importance from both a scientific and a practical perspective.

Domestication, the process whereby animals genetically adapt to a life under human auspices (Price 1999), fundamentally changes their physiology and behavior. In general, domesticated animals are less fearful and more tolerant of many environmental challenges compared to their wild ancestors (Jensen and Andersson 2005). This includes modifications in the stress responses, for example, reduced HPA reactivity as shown in several species (Ericsson *et al.* 2014; Treidman and Levine 1969; Martin 1978; Woodward and Strange 1987). Domesticated animals are fully able to breed with their wild ancestors. This offers a powerful tool to dissect the genetic mechanisms involved, by mapping the segregation of traits involved in the stress response.

Stress can be defined as a state of threatened homeostasis, which leads to physiological and behavioral alterations (McEwen 2007). The stress response includes activation of the sympathetic nervous system (SNS) and the HPA axis [reviewed by Steckler (2001)]. While considered as a way to cope with challenging situations, long-term chronic stress may lead to pathophysiological consequences (McEwen 2007). The general pattern of the stress response is highly conserved and similar among vertebrates, although the intensity has been modified during domestication. Following an acute stress exposure, release of hypothalamic corticotropin-releasing hormone (CRH) and other peptides into the pituitary leads to increased transcription of proopiomelanocortin (*POMC*) and the release of adrenocorticotropic hormone (ACTH) into the bloodstream. The production and release of the main categories of stress hormones, catecholamines and glucocorticosteroids, take place in the adrenal medulla and cortex, respectively. Furthermore, the adrenal gland is the main source of many other steroids including PREG, DHEA, and aldosterone (Blas 2015), which are also affected by stress. Individual corticosterone responses are consistent when age and environment are comparable, with a heritability estimate of 0.25–0.35 in several avian species (Brown and Nestor 1974; Satterlee and Johnson 1988; Jenkins *et al.* 2014; Evans *et al.* 2006).

The molecular basis of the stress response can be dissected by means of genetic mapping, where QTL analysis takes the polygenic nature of the stress into account. Earlier studies have reported QTL for both anxiety-related behavior and physiological stress in mice (Henderson *et al.* 2004), rats (Albert *et al.* 2008; Solberg *et al.* 2006), and salmonids (Drew *et al.* 2007). Domestication offers a strong opportunity for this method, and domesticated animals have previously been successfully used to map the genetic architecture of various complex traits (Andersson and Georges 2004; Goddard and Hayes 2009). In the present study, we focus on chickens to unravel the genetic mechanisms involved in domestication-related modifications of the stress response. Chickens were domesticated from their ancestors, the Red Junglefowl, about 8000 yr ago (Tixier-Boichard *et al.* 2011), and are the most widespread food-producing animals on earth today.

Using an advanced intercross between domesticated White Leghorn egg layers and ancestral Red Junglefowl, we have previously reported QTL and eQTL for, *e.g.*, anxiety-related behavior (Wright *et al.* 2006, 2012; Schütz *et al.* 2002, 2004; Johnsson *et al.* 2016). Furthermore, previous studies have shown significant differences in the endocrinological stress responses, hypothalamus, and adrenal gene expression between Red Junglefowl and White Leghorn (Ericsson *et al.* 2014; Fallahsharoudi *et al.* 2015; Nätt *et al.* 2012). Hence, the aim of this study was to use QTL analysis in the same advanced intercross line to investigate the genetic architecture of the stress response modifications caused by domestication. To do so, we used combined phenotypic QTL mapping with eQTL mapping of relevant genes in the relevant tissues to search for overlapping genomic regions. This allows an efficient localization of potential candidate genes. As the final step, we looked for correlations between expression levels of the identified genes and hormone levels, which provided a final list of strong candidate genes.

## Materials and Methods

### Ethical statement

All experimental protocols were approved by Linköping Council for Ethical Licensing of Animal Experiments, ethical permit no 122-10. Experiments were carried out in accordance with the approved guidelines.

### Animals and sample collection

A total of 232 birds from the 12th generation of an intercross between domesticated White Leghorn and ancestral Red Junglefowl were used. The birds were kept until final sampling at 6 wk of age, at which point they were killed for tissue sampling. The original cross was previously described in detail by Schütz *et al.* (2002) and Wright *et al.* (2006). Briefly, the intercross was generated by crossing one Red Junglefowl male and three White Leghorn females to get 41 F1 and then 821 F2 animals. The subsequent generations were maintained at population sizes of more than 100 chickens per generation with designed pedigrees to minimize loss of genetic diversity. The animals of the present study were hatched in four batches from a total of 30 families. A total of 88 animals were used in the eQTL mapping (the same birds were also used for hormone measurement). The birds were kept in groups of around 80 birds in 4 m^2^ pens with *ad libitum* access to food and water. The weight and metatarsus length were measured at hatching and at 6 wk of age. Blood samples were collected at baseline levels and, after 15 min of restraint in a hanging net, using methods previously described in detail (Fallahsharoudi *et al.* 2015). The blood samples were kept on ice, and then centrifuged at 2000 rpm for 5 min to collect the plasma. The plasma samples were stored at −80° until analysis. Immediately after the second blood sampling, the animals were decapitated, the brain area containing the hypothalamus was dissected as described previously (Nätt *et al.* 2009), and the adrenal glands were removed. The tissue samples were snap frozen in liquid nitrogen and kept in a −80° freezer until further analysis.

### Hormonal analysis

Hormones were analyzed with Ultra-Performance Convergence Chromatography (UPC2; Waters ACQUITY UPC2, Milford, MA) hyphenated with tandem mass spectrometry (XEVO TQ-S, Milford, MA). The qualitative analysis was performed at 40° using an Acquity UPC2 BEH column (100 mm, 3.0 mm, 1.7 µm; Waters). DHEA, PREG, corticosterone, aldosterone, and the internal standards DHEA-d2 and CORT-d8 were purchased from Steraloids Inc. (Newport, RI). Methoxyamine hydrochloride, the internal standard PREG-13C2-d2, LC grade highest purity solvents and chemicals were obtained from Sigma-Aldrich (Stockholm, Sweden), unless otherwise stated. Steroid extraction and derivatization were performed according to a previous method (Fallahsharoudi *et al.* 2015). In brief, 100 µl of chicken plasma was mixed with the internal standard mixture. Steroids were extracted with tert-Butyl methyl ether, gently vortexed for 15 min, and then centrifuged. The collected supernatant was dried under a stream of nitrogen gas. The extracted steroids were derivatized into methoxyamine by incubation at 60° for 45 min; excess reagents were dried under nitrogen. The derivatives of steroids were reconstituted in methanol. Samples were kept at −20° prior to analysis by supercritical fluid chromatography. Fallahsharoudi *et al.* (2015) previously reported the detailed methodology and separation conditions. Quantification was achieved using an isotope dilution technique. Duplicate injections of each sample were performed and the average values were reported (CV < 20%).

### RNA extraction and gene expression

RNA was extracted from the frozen brain and adrenal tissues using an AllPrep DNA/RNA Mini Kit (QIAGEN, Germany), and the potential residual DNA was digested on-column using a RNase-Free DNase Set (QIAGEN) during extraction according to the manufacturer’s instructions. The RNA quality was checked with an Agilent 2100 Bioanalyzer and all the RIN values were above nine. The single-stranded cDNA was prepared using a Maxima First Strand cDNA Synthesis Kit for quantitative real-time PCR (RT-qPCR) (Thermo Fisher Scientific) using 2.5 µg total RNA as the template. The list of genes within the C.I. of all genome-wide significant QTL was overlapped with the list of genes that were previously found to be significantly expressed in the adrenal glands (Fallahsharoudi *et al.* 2015) or the hypothalamus (Nätt *et al.* 2012) between White Leghorn and Red Junglefowl. Genes were then selected if they were located within the QTL C.I. and concurrently were differentially expressed in the adrenal or hypothalamus of White Leghorn and Red Junglefowl, as previously measured using microarrays. For corticosterone QTL on chromosome 5, six genes were measured in the adrenal gland, and for the aldosterone QTL, expression levels of one gene in the hypothalamus and eight genes in the adrenal gland were measured. We also measured expression levels of seven genes in the adrenal glands DHEA QTL. The list of all measured genes as well as their genomic location is presented in Supplemental Material, Table S1. Specific primers were designed using primer3 (Untergasser *et al.* 2012) and were blasted against the chicken genome using the NCBI primer blast tool to avoid multiple amplification. If possible, the primers were selected to be on exon/intron boundaries to evade amplification of potential residual genomic DNA. The specificity of primers was confirmed by observing a single band of PCR product by gel electrophoresis and by inspecting the melting curve in a Light Cycler. The reactions were performed in a Light Cycler 480 (Roche Diagnostics, Basel, Switzerland). Each reaction included 2 µl water, 1 µl of each forward and reverse primers (0.5 µM), 125 pg cDNA diluted in 1 µl water, and 5 µl SYBR Green I Master (Roche Diagnostics). The following RT-PCR reaction was performed: 5 min at 95° activation followed by 45 cycles of 10 sec at 95°, 10 sec at 60°, and 20 sec at 72°. The crossing point values were normalized over TATA box binding protein (TBP) as housekeeper. The relative expression difference between the individuals was measured according to the method developed by Pfaffl (2001).

### Genetic map and QTL analysis

DNA was extracted from the blood samples using a DNeasy Blood & Tissue Kit (QIAGEN) according to the manufacturer’s instructions. Genotyping was performed by Illumina Golden Gate in Uppsala sequencing and SNP platform. To construct the genetic map with a total length of 18,500 cM and average spacing of 26 cM, 739 genetic markers were used. Map construction and QTL mapping was conducted with R/qtl (Broman *et al.* 2003). SNPs were selected based on a previously obtained panel of 10,000 SNPs on parental birds and all used SNPs were fixed in F0. The map size in F2 was ∼3000 cM and was expanded to ∼9000 cM in the F8. Theoretically (Darvasi and Soller 1995), the map expansion is within expectation for an F12 generation considering the length of the F2 map. To detect single QTL, we examined the data with interval mapping and Haley–Knott regression (Haley and Knott 1992). Interval mapping was performed using additive and additive + dominance models. Plasmic levels of steroids and the normalized gene expression values were treated as quantitative phenotypes. Sex and batch were included in every analysis as fixed effects. To deal with the family structure in advanced intercross, at first a principal component analysis (PCA) of the genotype matrix was performed and the 10 strongest principal components (PC) were included in each regression model as covariates, and were tested for significance (Wu *et al.* 2011). All significant PCs were included in the final model. The significance thresholds for each trait were calculated with permutation tests, using 1000 permutations based on the full F12 map for each trait and model. A genome-wide 20% *p*-value cut-off was used for suggestive QTL, and *p*-values ≤ 5% were reported as significant QTL (Center 1995). For each QTL, 95% C.I. were calculated with a 1.8 LOD drop method (*i.e.*, where the LOD score on each side of the peak decreases by 1.8 LOD) (Manichaikul *et al.* 2006). The C.I. are extended to the closest marker to the 1.8 LOD drop. The physical location of the closest marker to the 95% C.I. for each QTL is reported in the chicken genome (galGal4 genome assembly). To analyze the effect of gene expression on hormone levels, a regression model with corticosterone response as dependent variable and transcription levels of measured genes as fixed factor was fitted (corticosterone response ∼ transcription levels + sex + batch + relatedness). The *p*-values were adjusted for multiple testing for all regression tests, with FDR correction. To further support the argument of causality, we fitted a regression model between gene expression and hormone levels and used genotypes at the nearest pseudomarker (obtained by using function makeqtl in the rqtl package) as a covariate in the model (transcription ∼ hormone + QTL+ sex + batch + relatedness). If the QTL marker has the main effect on both gene expression and hormonal response, we can expect to get nonsignificant or diminished estimates for the regression between hormones and gene expression, and the QTL marker should remain as the major model predictor.

### Data availability

Table S1 and Table S2 contain QTL statistics, and the centimorgan and physical location of all QTL and eQTL. File S1 includes genotypes for all markers and the measured phenotypes. File S2 contains the ID and physical location of all markers in the chicken genome (galGal4 genome assembly). 

## Results

**Figure 1:**
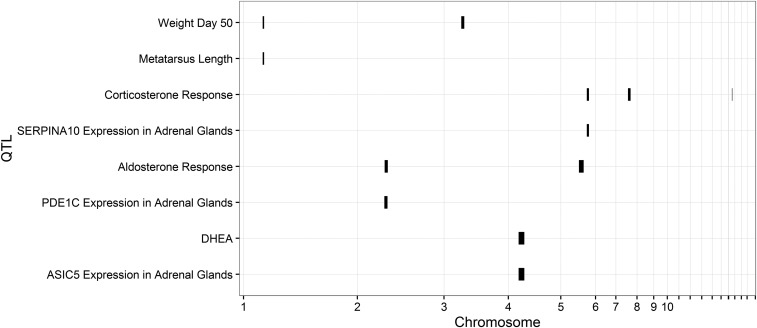
Genomic location of all detected QTL and eQTL. The width of the bars represent 95% C.I. obtained by 1.8 LOD drop. The C.I. are extended to the closest markers. The physical locations of markers were used to draw the figure. The relevant statistics are provided in Table S1 and Table S2. DHEA, dehydroepiandrosterone; eQTL, expression quantitative trait loci; LOD, logarithm of the odds; QTL, quantitative trait loci.

In crosses between ancestral Red Junglefowl and domesticated White Leghorn chickens, we used brief physical restraint as an acute stressor and measured the baseline and postrestraint levels of a number of steroids using tandem mass spectrometry. The experiment comprised 232 birds from the F12 generation of an advanced intercross, genotyped on 739 genetic markers throughout the genome. We mapped both the QTL associated with variations in hormone levels (Figure 1 and Table S2), as well as the QTL coupled to expression levels of the subset of genes that were located in the hormone QTL and that were also significantly differentially expressed in the adrenal glands or hypothalamus of the parental White Leghorn and Red Junglefowl at the same time (Table S1). The gene expression analysis was conducted on 88 genotyped birds. By overlaying the hormone level QTL with the eQTL, we identified loci associated with both. We then analyzed the correlations between the expression levels of candidate genes and the hormone responses. Significant correlation coupled with colocalization of QTL and eQTL was interpreted as a strong indication that we had identified a likely causative gene. The raw *p*-values were Bonferroni adjusted to correct for multiple testing for all pairwise tests conducted for each region (Table S1).

We found a QTL with large additive effects on both body weight and metatarsus length on chromosome 1, 34,741,424 (location of the closest marker to the QTL peak), and another with additive effects on body weight on chromosome 3, 29,811,624 (Figure 1 and Table S2). These QTL were situated at the same positions as previously reported (Wright *et al.* 2010; Henriksen *et al.* 2016). A significant QTL for corticosterone response (difference between post- and prerestraint) was detected on chromosome 5, and suggestive QTL for the same trait were found on chromosomes 7 and 17. Together, these three QTL explained 22.9% of the variance in corticosterone response (Figure 1 and Table S2). The QTL on chromosome 5 and 7 both had dominance effects, with the QTL on chromosome 7 having increased response levels associated with the White Leghorn allele (Table S2). The QTL on chromosome 17 showed higher response associated with the Red Junglefowl allele, as well as an interaction between genotype and sex. No significant QTL was detected for baseline levels of corticosterone or PREG. Furthermore, a significant QTL for baseline levels of DHEA was detected on chromosome 4 (explaining 4.9% of the variance), and two significant QTL were detected for postrestraint levels of aldosterone on chromosomes 2 and 5, together explaining 19.3% of the variance in stress-induced levels (Figure 1 and Table S2).

**Figure 2:**
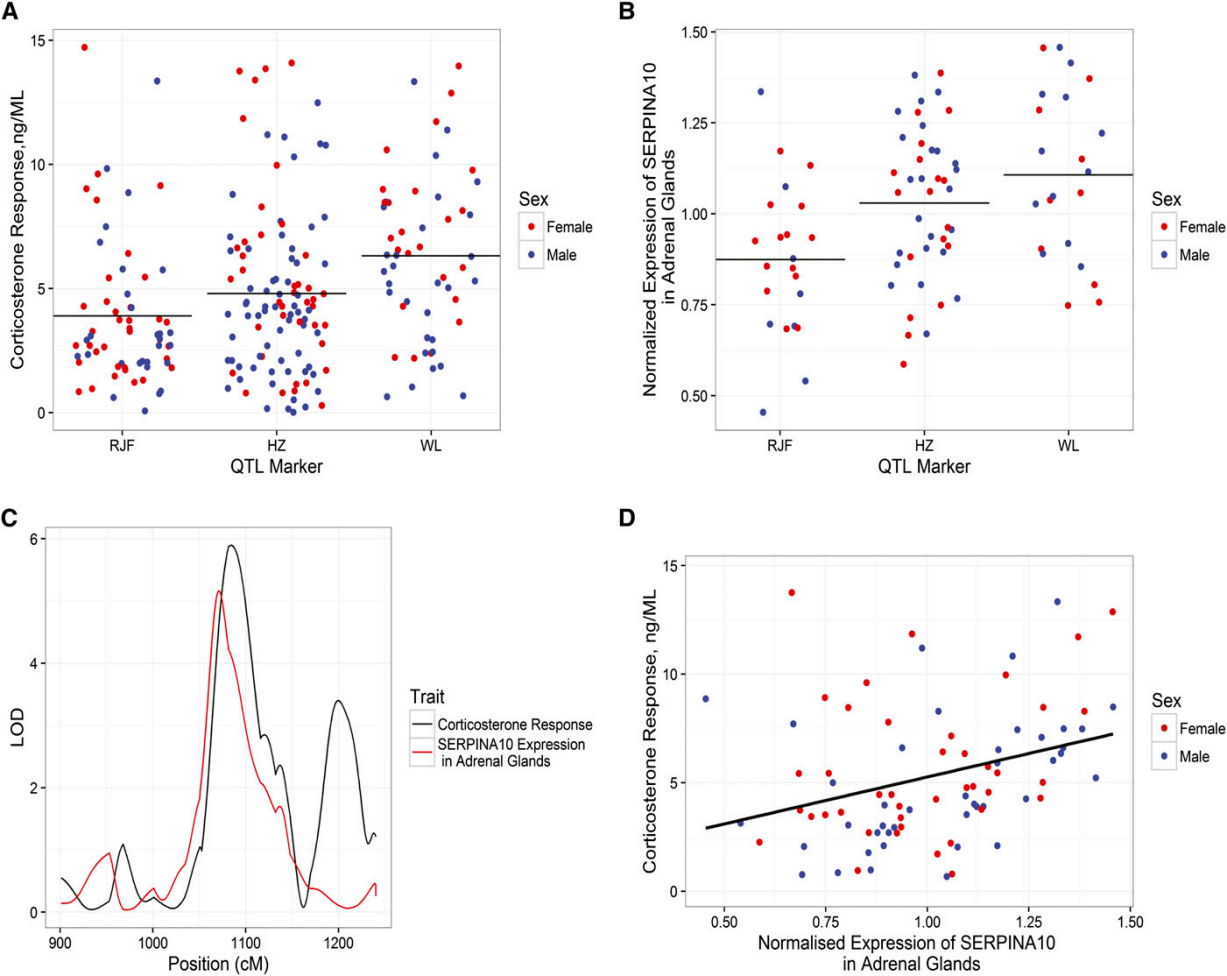
Genotype effect on transcription and hormone level. Effect of genotype of the closest marker to the corticosterone QTL peak (chromosome 5: 48,303,445) on the plasma levels of corticosterone (A) and on expression levels of *SERPINA10* in the adrenal glands (B). Bars in (A) and (B) represent the mean. RJF represent the homozygous locus for the Red Junglefowl allele, WL represents the homozygous locus for the White Leghorn allele, and HZ represents the heterozygous locus. Partial LOD curves of corticosterone response and *SERPINA 10* expression in adrenal glands (C). Linear relation between expression levels of *SERPINA10* in adrenal glands and plasma levels of corticosterone (D). LOD, logarithm of the odds; QTL, quantitative trait loci.

As a further attempt to identify candidate genes, we examined the genome region covered by the C.I. of all significant QTL for corticosterone response, aldosterone, and DHEA. Each region contained a total of 35–95 genes (Table S1). We compared the gene list to the findings of our previous experiments (Fallahsharoudi *et al.* 2015; Nätt *et al.* 2014), where we used microarrays to identify genes differentially expressed in the brain and the adrenal tissue when comparing purebred Red Junglefowl with White Leghorn. There was only one overlapping gene in the brain, but several genes appeared on the list of differentially expressed genes in the adrenal glands (Table S1) for each QTL. We measured the expression of 22 overlapping genes in the adrenal glands or brain using RT-qPCR, and then performed eQTL analysis for each of them. Of the six candidate genes underlying corticosterone response on chromosome 5, only *SERPINA10* had a significant eQTL located in the region examined. In addition, the expression of this gene correlated significantly with the corticosterone response (*r* = 0.32 and *p* = 0.003) (Figure 2 and Table S1). Next, we fitted a regression model between gene expression and corticosterone with the QTL marker as a covariate. The estimated effects for the QTL were still significant (*r*^2^ = 0.13 and *p* = 0.0006), but the estimated effect for the regression between gene expression and corticosterone was no longer significant (*r*^2^ = 1.2 and *p* = 0.2).

**Figure 3:**
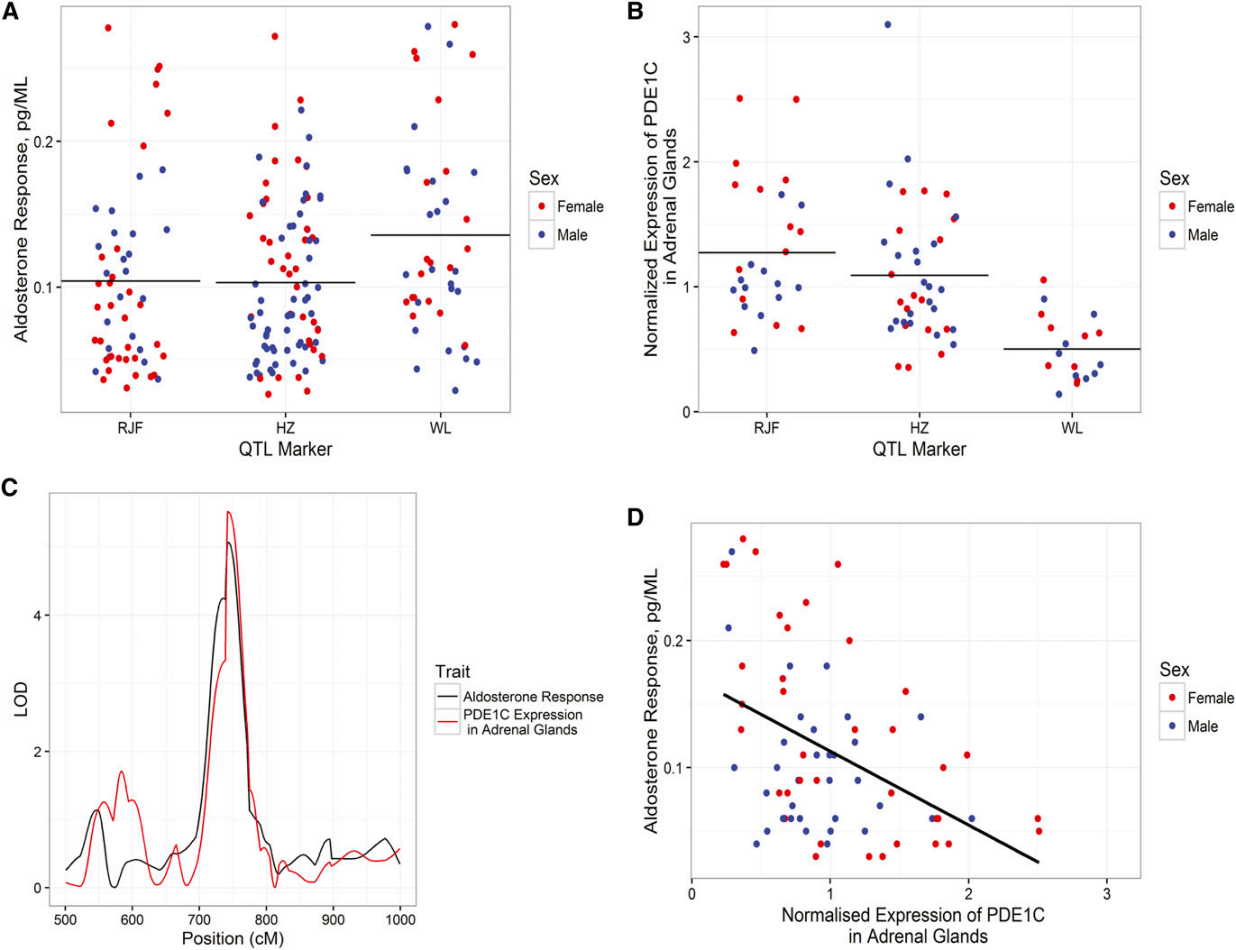
Genotype effect on transcription and hormone level. Effect of genotype of the closest marker to aldosterone QTL peak (chromosome 2: 48,799,369) on the plasma levels of aldosterone (A) and on expression levels of *PDE1C* in adrenal glands (B). Bars in (A) and (B) represent the mean. RJF represents the homozygous locus for the Red Junglefowl allele, WL represents the homozygous locus for the White Leghorn allele, and HZ represents the heterozygous locus. Partial LOD curves of corticosterone response and *PDE1C* expression in adrenal glands (C). Correlations between expression levels of *PDE1C* in adrenal glands and plasma levels of aldosterone (D). LOD, logarithm of the odds; QTL, quantitative trait loci.

Next, we applied the same method to identify the candidate gene(s) underlying the QTL for aldosterone and DHEA. Three potential candidate genes for the aldosterone QTL on chromosome 2 were found in the list of differentially expressed genes in the brain or adrenal glands (two in adrenal glands and one in brain). Of these three candidate genes, only phosphodiesterase 1C (*PDE1C*) had a significant eQTL. The expression of *PDE1C* in adrenal glands was also significantly correlated with the aldosterone levels (*r* = −0.27 and *p* = 0.01) (Figure 3 and Table S1). Then, a regression model between gene expression levels of *PDE1C* and aldosterone with the QTL marker as a covariate was fitted. The estimated effects for the QTL were the main model predictors (*r*^2^ = 0.22 and *p* = 7.5 e^−6^), and the estimated effect for the regression between gene expression and aldosterone was also significant (*r*^2^ = 0.12 and *p* = 0.0003).

We measured adrenal expression of six potential candidate genes for the aldosterone QTL on chromosome 5, but none of the measured genes showed overlapping eQTL. Among the six candidate genes in the QTL for DHEA on chromosome 4, acid-sensing ion channel subunit family member 5 (*ASIC5*) was the only gene with a significant overlapping eQTL, but the expression of *ASIC5* was not correlated with plasma levels of DHEA.

## Discussion

Our results show that domestication has modified the stress response in chickens by changes in parts of the genome involved in the control of the HPA axis. In particular, we suggest that the genes *SERPINA10* and *PDE1C* are strong candidates for this effect, although our present results do not allow us to pinpoint any potential causal mutations. The fact that the genes are differentially expressed when ancestral Red Junglefowl is compared to domesticated White Leghorn, and the fact that the gene expression effect can be mapped back to the physical location of the genes, indicates that the effects observed on corticosterone reactivity is most likely caused by mutations in the regulatory regions of the gene. This represents the first identification of putative causative genes involved in the domestication of the stress response in chickens.

Previously, we have shown that domesticated chickens have a less pronounced acute corticosterone response than their progenitors, but take longer to recover (Ericsson *et al.* 2014). This was also the case when comparing behavioral reactions. In the present study, we detected one significant and two suggestive QTL for the corticosterone response. However, the direction of the effect for the significant QTL for the corticosterone response was opposite to that seen in the purebred birds, in the sense that the White Leghorn allele was associated with higher levels of corticosterone response. The occurrence of QTL with opposite effects in intercrosses is observed occasionally, and is often a consequence of recombination between lines that are fixed for a set of alleles with opposite direction within each line (Rieseberg *et al.* 1999, 2003).

In general, colocalization of expression QTL and phenotypic QTL, accompanied by correlation between gene expression and phenotype, is regarded as strong evidence that the candidate gene is causing the QTL (Doss *et al.* 2005). Our results suggest that the *SERPINA10* gene is the most probable candidate gene underlying the corticosterone response QTL on chromosome 5. This gene was the only one of the candidates with an adrenal eQTL overlapping the corticosterone QTL, and at the same time the relative expression of the gene was correlated to the levels of corticosterone in plasma.

Serpins (serine protease inhibitors) are proteins with many functions, including hormone transport, protease inhibition, and involvement in secretory pathways. In mammals, *SERPIN1* and *SERPIN10* are suggested to be involved in protease inhibition and the regulation of secretory pathways (Ragg 2007; Thomas 2002). In chickens, *SERPINA10* is located in a region containing a cluster of related clade A *SERPIN* genes, with homologous regions in other vertebrates (Forsyth *et al.* 2003; Vashchenko *et al.* 2016). QTL in this region have previously been reported for corticosterone response in rats and pigs (Solberg *et al.* 2006; Désautés *et al.* 2002). In both these species, *SERPIN6*, which codes corticotrophin-binding globulin (CBG), was speculated to be the main candidate gene based on function (Breuner and Orchinik 2002). In birds, although the CBG protein is found in plasma (Breuner and Orchinik 2002), the CBG gene is not annotated, but it has been suggested that *SERPIN4* is homologous with *SERPINA6* in chicken (Vashchenko *et al.* 2016). It is also striking that *SERPINA10* is located very close to a selective sweep associated with domestication in chickens (Rubin *et al.* 2010), which is in-line with the suggestion that the gene has been important in modifying the stress reaction during domestication.

Of the other seven genes located within the C.I. of the corticosterone QTL on chromosome 5, three have a known biological function, which makes them potential candidates as well. *VRK1* is an early stress response gene belonging to the family of serine–threonine kinases. *VRK1* is involved in the phosphorylation of transcription factors related to the stress response. This phosphorylation leads to accumulation of c-Jun and c-Fos proteins in the cell, which represents a central signaling pathway (Valbuena *et al.* 2008; Sevilla *et al.* 2004). A second potential candidate, bradykinin B2 Receptor (*BDKRB2*), mediates the effect of bradykinin during the stress response and is involved in catecholamine biosynthesis. Nostramo *et al.* (2013) showed that the expression levels of *BDKRB2* increase in the adrenal medulla of rats after restraint stress. The third potential candidate is *AK7*, which is a member of the adenylate kinase family. These three genes are also found in QTL regions for HPA-activity in rats (Solberg *et al.* 2006) and pigs (Désautés *et al.* 2002), suggesting that this genetic region mediates stress reactivity in several vertebrates. Although none of these genes had eQTL coinciding with a corticosterone QTL, and none showed a significant correlation between expression and corticosterone levels, they may be of central importance for the stress response and may, for example, play a part in modulation of *SERPINA10* expression in adrenal glands.

There were two significant QTL on chromosome 2 and 5 related to postrestraint levels of aldosterone. This hormone increases in response to a variety of stressors including restraint (Stier *et al.* 2004; Aguilera *et al.* 1995) and has a crucial function in retaining salt and water balance during stress. We found one candidate gene for aldosterone response on chromosome 2, *PDE1C*. The main function of cyclic AMP (cAMP) phosphodiesterases (PDEs) is to degrade cAMP, a widespread second messenger (Tsai and Beavo 2012). The cAMP/protein kinase A (cAMP/PKA) pathway is the central signaling pathway in the regulation of StAR activity and steroidogenesis. The level of cAMP in cells is determined by its synthesis rate via adenylyl cyclases (ACs), as well as its degradation rate via PDEs. cAMP PDEs are involved in the regulation of steroidogenesis by modulation of the cAMP/PKA transduction pathway (Stocco *et al.* 2005; Cherradi *et al.* 2003). Multiple PDE isoenzymes with specific functions, cell type expression, and regulation are recognized (Beavo *et al.* 1994). PDEs can also have inhibitory effects on the cAMP-dependent PKA transduction pathway and, hence steroidogenesis by degradation of cellular cAMP (Stocco *et al.* 2005; Beavo *et al.* 1994).

We were unable to identify any candidate gene for aldosterone on chromosome 5. The QTL may thus be caused by protein changing mutation(s) or DNA variants influencing post-transcriptional mechanisms. Several studies have reported protein-specific QTL with no effect on mRNA abundance [reviewed by Albert and Kruglyak (2015)]. Another possibility is that a DNA variant in the aldosterone QTL on chromosome 5 may contain *trans*-regulatory elements that affect the aldosterone levels by modifying the expression of one or several distant genes.

We also discovered a significant QTL for the levels of DHEA, which is suggested to be involved in aggression and social behaviors in birds (Soma *et al.* 2002; Genud *et al.* 2008; Boonstra *et al.* 2008). Among the potential candidate genes underlying the QTL, *ASIC5* had a significant overlapping eQTL, but no correlation was found between *ASIC5* expression in the adrenal glands and DHEA. Hence, we were not able to identify any strong candidate for this hormone.

In conclusion, we found several QTL regions involved in modifications of the stress response that have occurred during chicken domestication. Our results specifically suggest that *SERPINA10* and *PDE1C* may be strong candidate genes affecting the differences between ancestral Red Junglefowl and domesticated White Leghorn chickens.

## Supplementary Material

Supplemental material is available online at www.g3journal.org/lookup/suppl/doi:10.1534/g3.116.037721/-/DC1.

## References

[bib1] Aguilera G. Kiss A. Luo X. U. N. Akbasak B.-S., 1995 The renin angiotensin system and the stress response. Ann. N. Y. Acad. Sci. 771(1): 173–186. 8597396 10.1111/j.1749-6632.1995.tb44679.x

[bib2] Albert F. W. Kruglyak L., 2015 The role of regulatory variation in complex traits and disease. Nat. Rev. Genet. 16(4): 197–212. 25707927 10.1038/nrg3891

[bib3] Albert F. W. Shchepina O. Winter C. Römpler H. Teupser D., 2008 Phenotypic differences in behavior, physiology and neurochemistry between rats selected for tameness and for defensive aggression towards humans. Horm. Behav. 53(3): 413–421. 18177873 10.1016/j.yhbeh.2007.11.010

[bib4] Andersson L. Georges M., 2004 Domestic-animal genomics: deciphering the genetics of complex traits. Nat. Rev. Genet. 5(3): 202–212. 14970822 10.1038/nrg1294

[bib5] Beavo J. A. Conti M. Heaslip R. J., 1994 Multiple cyclic nucleotide phosphodiesterases. Mol. Pharmacol. 46(3): 399–405. 7935318

[bib6] Blas J., 2015 Chapter 33 - Stress in birds A2 - Scanes, pp. 769–810 in Sturkie’s *Avian Physiology*, Ed. 6, edited by Colin G. Academic Press, San Diego.

[bib7] Boonstra R. Lane J. E. Boutin S. Bradley A. Desantis L., 2008 Plasma DHEA levels in wild, territorial red squirrels: seasonal variation and effect of ACTH. Gen. Comp. Endocrinol. 158(1): 61–67. 18558404 10.1016/j.ygcen.2008.05.004

[bib8] Breuner C. Orchinik M., 2002 Plasma binding proteins as mediators of corticosteroid action in vertebrates. J. Endocrinol. 175(1): 99–112. 12379494 10.1677/joe.0.1750099

[bib9] Broman K. W. Wu H. Sen Ś. Churchill G. A., 2003 R/qtl: QTL mapping in experimental crosses. Bioinformatics 19(7): 889–890. 12724300 10.1093/bioinformatics/btg112

[bib10] Brown K. I. Nestor K. E., 1974 Implications of selection for high and low adrenal response to stress. Poult. Sci. 53(4): 1297–1306. 4368795 10.3382/ps.0531297

[bib11] Center C., 1995 Genetic dissection of complex traits: guidelines for interpreting and reporting linkage results. Nat. Genet. 11: 241–247. 7581446 10.1038/ng1195-241

[bib12] Cherradi N. Pardo B. Greenberg A. S. Kraemer F. B. Capponi A. M., 2003 Angiotensin II activates cholesterol ester hydrolase in bovine adrenal glomerulosa cells through phosphorylation mediated by p42/p44 mitogen-activated protein kinase. Endocrinology 144(11): 4905–4915. 12960096 10.1210/en.2003-0325

[bib13] Darvasi A. Soller M., 1995 Advanced intercross lines, an experimental population for fine genetic mapping. Genetics 141(3): 1199–1207. 8582624 10.1093/genetics/141.3.1199 PMC1206841

[bib14] Désautés C. Bidanel J. Milan D. Iannuccelli N. Amigues Y., 2002 Genetic linkage mapping of quantitative trait loci for behavioral and neuroendocrine stress response traits in pigs. J. Anim. Sci. 80(9): 2276–2285. 12350005 10.2527/2002.8092276x

[bib15] Doss S. Schadt E. E. Drake T. A. Lusis A. J., 2005 *Cis*-acting expression quantitative trait loci in mice. Genome Res. 15(5): 681–691. 15837804 10.1101/gr.3216905 PMC1088296

[bib16] Drew R. E. Schwabl H. Wheeler P. A. Thorgaard G. H., 2007 Detection of QTL influencing cortisol levels in rainbow trout (*Oncorhynchus mykiss*). Aquaculture 272: S183–S194.

[bib17] Ericsson M. Fallahsharoudi A. Bergquist J. Kushnir M. M. Jensen P., 2014 Domestication effects on behavioural and hormonal responses to acute stress in chickens. Physiol. Behav. 133: 161–169. 24878317 10.1016/j.physbeh.2014.05.024

[bib18] Evans M. R. Roberts M. L. Buchanan K. L. Goldsmith A. R., 2006 Heritability of corticosterone response and changes in life history traits during selection in the zebra finch. J. Evol. Biol. 19(2): 343–352. 16599910 10.1111/j.1420-9101.2005.01034.x

[bib19] Fallahsharoudi A. de Kock N. Johnsson M. Ubhayasekera S. J. Bergquist J., 2015 Domestication effects on stress induced steroid secretion and adrenal gene expression in chickens. Sci. Rep. 5: 15345. 26471470 10.1038/srep15345 PMC4608001

[bib20] Forsyth S. Horvath A. Coughlin P., 2003 A review and comparison of the murine α_1_-antitrypsin and α_1_-antichymotrypsin multigene clusters with the human clade A serpins. Genomics 81(3): 336–345. 12659817 10.1016/s0888-7543(02)00041-1

[bib21] Genud R. Merenlender A. Gispan-Herman I. Maayan R. Weizman A., 2008 DHEA lessens depressive-like behavior via GABA-ergic modulation of the mesolimbic system. Neuropsychopharmacology 34(3): 577–584. 18496525 10.1038/npp.2008.46

[bib22] Goddard M. E. Hayes B. J., 2009 Mapping genes for complex traits in domestic animals and their use in breeding programmes. Nat. Rev. Genet. 10(6): 381–391. 19448663 10.1038/nrg2575

[bib23] Haley C. S. Knott S. A., 1992 A simple regression method for mapping quantitative trait loci in line crosses using flanking markers. Heredity 69(4): 315–324. 16718932 10.1038/hdy.1992.131

[bib24] Henderson N. D. Turri M. G. DeFries J. C. Flint J., 2004 QTL analysis of multiple behavioral measures of anxiety in mice. Behav. Genet. 34(3): 267–293. 14990867 10.1023/B:BEGE.0000017872.25069.44

[bib25] Henriksen R. Johnsson M. Andersson L. Jensen P. Wright D., 2016 The domesticated brain: genetics of brain mass and brain structure in an avian species. Sci. Rep. 6: 34031. 27687864 10.1038/srep34031 PMC5043184

[bib26] Jenkins B. R. Vitousek M. N. Hubbard J. K. Safran R. J., 2014 An experimental analysis of the heritability of variation in glucocorticoid concentrations in a wild avian population. Proc. Biol. Sci. 281: 20141302. 25056627 10.1098/rspb.2014.1302 PMC4123711

[bib27] Jensen P. Andersson L., 2005 Genomics meets ethology: a new route to understanding domestication, behavior, and sustainability in animal breeding. Ambio 34(4–5): 320–324. 16092263 10.1639/0044-7447(2005)034[0320:gmeanr]2.0.co;2

[bib28] Johnsson M. Williams M. J. Jensen P. Wright D., 2016 Genetical genomics of behavior: a novel chicken genomic model for anxiety behavior. Genetics 202(1): 327–340. 26733665 10.1534/genetics.115.179010 PMC4701096

[bib29] Lupien S. J. McEwen B. S. Gunnar M. R. Heim C., 2009 Effects of stress throughout the lifespan on the brain, behaviour and cognition. Nat. Rev. Neurosci. 10(6): 434–445. 19401723 10.1038/nrn2639

[bib30] Manichaikul A. Dupuis J. Sen Ś. Broman K. W., 2006 Poor performance of bootstrap confidence intervals for the location of a quantitative trait locus. Genetics 174(1): 481–489. 16783000 10.1534/genetics.106.061549 PMC1569776

[bib31] Martin J. T., 1978 Embryonic pituitary adrenal axis, behavior development and domestication in birds. Am. Zool. 18(3): 489–499.

[bib32] McEwen B. S., 2007 Physiology and neurobiology of stress and adaptation: central role of the brain. Physiol. Rev. 87(3): 873–904. 17615391 10.1152/physrev.00041.2006

[bib33] Nätt D. Lindqvist N. Stranneheim H. Lundeberg J. Torjesen P. A., 2009 Inheritance of acquired behaviour adaptations and brain gene expression in chickens. PLoS One 4(7): e6405. 19636381 10.1371/journal.pone.0006405 PMC2713434

[bib34] Nätt D. Rubin C.-J. Wright D. Johnsson M. Beltéky J., 2012 Heritable genome-wide variation of gene expression and promoter methylation between wild and domesticated chickens. BMC Genomics 13(1): 1–12. 22305654 10.1186/1471-2164-13-59 PMC3297523

[bib35] Nätt D. Agnvall B. Jensen P., 2014 Large sex differences in chicken behavior and brain gene expression coincide with few differences in promoter DNA-methylation. PLoS One 9(4): e96376. 24782041 10.1371/journal.pone.0096376 PMC4004567

[bib36] Nostramo R. Tillinger A. Serova L. Kvetnansky R. Sabban E. L., 2013 Bradykinin B2 receptor in the adrenal medulla of male rats and mice: glucocorticoid-dependent increase with immobilization stress. Endocrinology 154(10): 3729–3738. 24025224 10.1210/en.2013-1406

[bib37] Pfaffl M. W., 2001 A new mathematical model for relative quantification in real-time RT–PCR. Nucleic Acids Res. 29(9): e45. 11328886 10.1093/nar/29.9.e45 PMC55695

[bib38] Price E. O., 1999 Behavioral development in animals undergoing domestication. Appl. Anim. Behav. Sci. 65(3): 245–271.

[bib39] Ragg H., 2007 The role of serpins in the surveillance of the secretory pathway. Cell. Mol. Life Sci. 64(21): 2763–2770. 17668148 10.1007/s00018-007-7157-0 PMC11136186

[bib40] Rieseberg L. H. Archer M. A. Wayne R. K., 1999 Transgressive segregation, adaptation and speciation. Heredity 83(4): 363–372. 10583537 10.1038/sj.hdy.6886170

[bib41] Rieseberg L. H. Widmer A. Arntz A. M. Burke B., 2003 The genetic architecture necessary for transgressive segregation is common in both natural and domesticated populations. Philos. Trans. R. Soc. Lond. B. Biol. Sci. 358(1434): 1141–1147. 12831480 10.1098/rstb.2003.1283 PMC1693210

[bib42] Rostagno M. H., 2009 Can stress in farm animals increase food safety risk? Foodborne Pathog. Dis. 6(7): 767–776. 19737056 10.1089/fpd.2009.0315

[bib43] Rubin C.-J. Zody M. C. Eriksson J. Meadows J. R. S. Sherwood E., 2010 Whole genome resequencing reveals loci under selection during chicken domestication. Nature 464: 587–591. 20220755 10.1038/nature08832

[bib44] Satterlee D. Johnson W., 1988 Selection of Japanese quail for contrasting blood corticosterone response to immobilization. Poult. Sci. 67(1): 25–32. 3375175 10.3382/ps.0670025

[bib45] Schütz K. Kerje S. Carlborg Ö. Jacobsson L. Andersson L., 2002 QTL analysis of a red junglefowl × White Leghorn intercross reveals trade-off in resource allocation between behavior and production traits. Behav. Genet. 32(6): 423–433. 12467340 10.1023/a:1020880211144

[bib46] Schütz K. Kerje S. Jacobsson L. Forkman B. Carlborg Ö., 2004 Major growth QTLs in fowl are related to fearful behavior: possible genetic links between fear responses and production traits in a red junglefowl × White Leghorn intercross. Behav. Genet. 34(1): 121–130. 14739702 10.1023/B:BEGE.0000009481.98336.fc

[bib47] Sevilla A. Santos C. R. Barcia R. Vega F. M. Lazo P. A., 2004 c-Jun phosphorylation by the human vaccinia-related kinase 1 (VRK1) and its cooperation with the N-terminal kinase of c-Jun (JNK). Oncogene 23(55): 8950–8958. 15378002 10.1038/sj.onc.1208015

[bib48] Solberg L. C. Baum A. E. Ahmadiyeh N. Shimomura K. Li R., 2006 Genetic analysis of the stress-responsive adrenocortical axis. Physiol. Genomics 27(3): 362–369. 16895972 10.1152/physiolgenomics.00052.2006

[bib49] Soma K. K. Wissman A. M. Brenowitz E. A. Wingfield J. C., 2002 Dehydroepiandrosterone (DHEA) increases territorial song and the size of an associated brain region in a male songbird. Horm. Behav. 41(2): 203–212. 11855905 10.1006/hbeh.2001.1750

[bib50] Steckler T., 2001 The molecular neurobiology of stress–evidence from genetic and epigenetic models. Behav. Pharmacol. 12(6–7): 381–427. 11742135 10.1097/00008877-200111000-00002

[bib51] Stier C. T. Jr Serova L. I. Singh G. Sabban E. L., 2004 Stress triggered rise in plasma aldosterone is lessened by chronic nicotine infusion. Eur. J. Pharmacol. 495(2–3): 167–170. 15249166 10.1016/j.ejphar.2004.05.030

[bib52] Stocco D. M. Wang X. Jo Y. Manna P. R., 2005 Multiple signaling pathways regulating steroidogenesis and steroidogenic acute regulatory protein expression: more complicated than we thought. Mol. Endocrinol. 19(11): 2647–2659. 15831519 10.1210/me.2004-0532

[bib53] Thomas G., 2002 Furin at the cutting edge: from protein traffic to embryogenesis and disease. Nat. Rev. Mol. Cell Biol. 3(10): 753–766. 12360192 10.1038/nrm934 PMC1964754

[bib54] Tixier-Boichard M. Bed’hom B. Rognon X., 2011 Chicken domestication: from archeology to genomics. C. R. Biol. 334(3): 197–204. 21377614 10.1016/j.crvi.2010.12.012

[bib55] Treidman D. M. Levine S., 1969 Plasma corticosteroid response to stress in four species of wild mice. Endocrinology 84(3): 676–680. 4304265 10.1210/endo-84-3-676

[bib56] Tsai L. L. Beavo J., 2012 Regulation of adrenal steroidogenesis by the high-affinity phosphodiesterase 8 family. Horm. Metab. Res. 44(10): 790–794. 22903278 10.1055/s-0032-1321861 PMC4020523

[bib57] Untergasser A. Cutcutache I. Koressaar T. Ye J. Faircloth B. C., 2012 Primer3—new capabilities and interfaces. Nucleic Acids Res. 40(15): e115. 22730293 10.1093/nar/gks596 PMC3424584

[bib58] Valbuena A. López-Sánchez I. Lazo P. A., 2008 Human VRK1 is an early response gene and its loss causes a block in cell cycle progression. PLoS One 3(2): e1642. 18286197 10.1371/journal.pone.0001642 PMC2241669

[bib59] Vashchenko G. Das S. Moon K.-M. Rogalski J. C. Taves M. D., 2016 Identification of avian corticosteroid-binding globulin (SerpinA6) reveals the molecular basis of evolutionary adaptations in SerpinA6 structure and function as a steroid-binding protein. J. Biol. Chem. 291(21): 11300–11312. 27026706 10.1074/jbc.M116.714378 PMC4900275

[bib60] Woodward C. C. Strange R. J., 1987 Physiological stress responses in wild and hatchery-reared rainbow trout. Trans. Am. Fish. Soc. 116(4): 574–579.

[bib61] Wright D. Kerje S. Lundström K. Babol J. Schütz K., 2006 Quantitative trait loci analysis of egg and meat production traits in a red junglefowl × White Leghorn cross. Anim. Genet. 37(6): 529–534. 17121597 10.1111/j.1365-2052.2006.01515.x

[bib62] Wright D. Rubin C. J. Martinez Barrio A. Schütz K. Kerje S., 2010 The genetic architecture of domestication in the chicken: effects of pleiotropy and linkage. Mol. Ecol. 19(23): 5140–5156. 21040053 10.1111/j.1365-294X.2010.04882.x

[bib63] Wright D. Rubin C. Schutz K. Kerje S. Kindmark A., 2012 Onset of sexual maturity in female chickens is genetically linked to loci associated with fecundity and a sexual ornament. Reprod. Domest. Anim. 47(Suppl. 1): 31–36. 10.1111/j.1439-0531.2011.01963.x 22212210

[bib64] Wu C. DeWan A. Hoh J. Wang Z., 2011 A comparison of association methods correcting for population stratification in case–control studies. Ann. Hum. Genet. 75(3): 418–427. 21281271 10.1111/j.1469-1809.2010.00639.x PMC3215268

