# Supplementary material for: Genetic and Targeted eQTL Mapping Reveals Strong Candidate Genes Modulating the Stress Response During Chicken Domestication
Source: G3 (Bethesda). 2016 Dec 10;7(2):497–504. doi: 10.1534/g3.116.037721 (PMC5295596; doi:10.1534/g3.116.037721)
Supplement: Supplementary file 4 [file 497FileS2.docx]

**File S2** Marker Locations. (.csv, 21 KB)

Available for download as a .csv file at

<http://www.g3journal.org/lookup/suppl/doi:10.1534/g3.116.037721/-/DC1/FileS2.csv>
